# Potential impact of propofol immediately after motor vehicle accident on later symptoms of posttraumatic stress disorder at 6-month follow up: a retrospective cohort study

**DOI:** 10.1186/cc11681

**Published:** 2012-10-28

**Authors:** Masato Usuki, Yutaka Matsuoka, Daisuke Nishi, Naohiro Yonemoto, Kenta Matsumura, Yasuhiro Otomo, Yoshiharu Kim, Shigenobu Kanba

**Affiliations:** 1 National Institute of Mental Health, National Center of Neurology and Psychiatry, Tokyo 187-8553, Japan; 2 Department of Psychiatry, National Disaster Medical Center, Tokyo 190-0014, Japan; 3 CREST, Japan Science and Technology Agency, Tokyo 102-0075, Japan; 4 Department of Neuropsychiatry, Graduate School of Medical Science, Kyushu University, Fukuoka 812-8582, Japan; 5 Department of Clinical Epidemiology, Translational Medical Center, National Center of Neurology and Psychiatry, Tokyo 187-8551, Japan; 6 Department of Acute Critical Care and Disaster Medicine, Tokyo Medical and Dental University, Tokyo 113-8510, Japan

## Abstract

**Introduction:**

Critically injured patients are at risk of developing posttraumatic stress disorder (PTSD). Propofol was recently reported to enhance fear memory consolidation retrospectively. Thus, we investigated here whether administration of propofol within 72 h of a motor vehicle accident (MVA) affects the subsequent development of PTSD symptoms.

**Methods:**

We examined data obtained from a prospective cohort study of MVA-related injured patients, admitted to the intensive care unit of a general hospital. We investigated the effect of propofol administration within 72 h of MVA on outcome. Primary outcome was diagnosis of full or partial PTSD as determined by the Clinician-Administered PTSD Scale (CAPS) at 6 months. Secondary outcomes were diagnosis of full or partial PTSD at 1 month and CAPS score indicating PTSD at 1 and 6 months. Multivariate analysis was conducted adjusting for being female, age, injury severity score (ISS), and administration of ketamine or midazolam within 72 h of MVA.

**Results:**

Among 300 patients recruited (mean ISS, 8.0; median Glasgow Coma Scale (GCS) score, 15.0; age, 18 to 69 years), propofol administration showed a higher risk for full or partial PTSD as determined by CAPS at 6 months (odds ratio = 6.13, 95% confidence interval (CI): 1.57 to 23.85, P = 0.009) and at 1 month (odds ratio = 1.31, 95% CI: 0.41 to 4.23, P = 0.647) in the multivariate logistic regression. Multivariate regression analysis showed a trend toward adverse effects of propofol on PTSD symptom development at 6 months after MVA (β = 4.08, 95% CI: -0.49 to 8.64, P = 0.080), but not at 1 month after MVA (β = -0.42, 95% CI: -6.34 to 5.51, P = 0.890).

**Conclusions:**

These findings suggest that using propofol in the acute phase after MVA might be associated with the development of PTSD symptoms 6 months later. However, since the design of this study was retrospective, these findings should be interpreted cautiously and further study is warranted.

## Introduction

Critically injured patients are at risk of developing posttraumatic stress disorder (PTSD), particularly those injured in a motor vehicle accident (MVA) [1-10]. MVA survivors with psychiatric morbidity such as PTSD have also been found to have significantly lower quality of life and post-accident work potential than those without psychiatric morbidity [11,12]. Thus, it is important to detect MVA survivors at risk of developing later PTSD and prevent it when feasible.

In general, MVA survivors are administered sedatives for agitation while in the intensive care unit (ICU) or in perioperative management after a traumatic experience. The American College of Critical Care Medicine, clinical practice guidelines recommend use of intravenous propofol, midazolam, or lorazepam for sustaining sedation in the ICU [13]. Some researchers have examined whether sedative drugs affect memory function and the subsequent development of PTSD symptoms. Fisher *et al. *reported that midazolam exposure resulted in antegrade memory loss in humans [14]. McGee *et al.*, working on the assumption that midazolam decreases fear memory, examined the effectiveness of midazolam for later PTSD, but found the prevalence of PTSD did not differ between injured soldiers who received the drug intraoperatively, and those who did not [15].

Recently, Hauer *et al. *reported that retrospectively propofol enhances consolidation of fear memory in rats [16]. Their experiment, using a well-characterized animal model of aversive training, showed results similar to those for MVA survivors who were administered propofol during acute trauma care and later presented with PTSD. It is speculated that the core mechanism behind the development of PTSD is excessive consolidation of and failure to extinguish fear memory; therefore, modulating consolidation of fear memory as part of early intervention after a traumatic experience would be a potential strategy to prevent PTSD symptoms from developing later [17]. However, the findings of Hauer *et al. *were obtained in an animal model, and it is still unclear whether administration of propofol for MVA survivors in the clinical setting is related to later PTSD development.

We hypothesized that administration of propofol at an early stage after a traumatic experience might increase the risk for PTSD symptoms later, and investigated here whether such administration within 72 h of MVA is associated with the development of PTSD symptoms 6 months later.

## Materials and methods

### Participants

#### Overview of the Tachikawa cohort of the Motor Vehicle Accident Study

This study was performed as a part of the Tachikawa cohort of the MVA (TCOM) Study [10], a prospective cohort study which was conducted in accordance with the Declaration of Helsinki and approved by the Ethics Committee of the National Disaster Medical Center (NDMC), Tokyo. NDMC serves a population of approximately 1.7 million and its acute critical care center is responsible for level-I trauma service provision. Participants in the present study were patients consecutively admitted to the ICU of NDMC with MVA-related injury between 30 May 2004 and 8 January 2008. During this period, 344 patients met the eligible criteria and were asked to participate in the study. After receiving a description of the study, 300 patients (87.2%) provided written informed consent. The median number of days between the time of MVA (confirmed from ambulance service records) and baseline assessments was 2.3 days (range 0 to 23).

#### Study participants

The inclusion criteria were as follows: i) MVA-related severe physical injury causing a life-threatening or critical condition; ii) consecutive admittance to the NDMC acute critical care center (ICU); iii) age between 18 and 69 years, and iv) native Japanese speaking ability. The exclusion criteria were as follows: i) diffuse axonal injury, brain contusion, or subdural or subarachnoidal bleeding detected by computed tomography and/or magnetic resonance imaging (with the exception of concussion), because the presence of traumatic brain injury creates considerable difficulties when assessing psychological responses to injury; ii) cognitive impairment, defined as a score < 24 on the Mini-Mental State Examination (MMSE); iii) currently suffering from schizophrenia, bipolar disorder, drug (non-alcohol) dependence or abuse, or epilepsy before the MVA; iv) marked serious symptoms such as suicidal ideation, self-harm behavior, dissociation, or a severe physical condition preventing the patient from tolerating the interview, and v) living or working at a location more than 40 km from the NDMC. Whether patients met the inclusion or exclusion criteria was clinically assessed by a trained research nurse or psychiatrist.

### Assessments

#### Baseline and follow-up assessments

The following baseline data were gathered: general socio-demographics, past history of psychopathology as determined in a structured interview, detailed information about the MVA, vital signs first recorded on admission to the emergency room, injury severity score (ISS) [18], Glasgow Coma Scale (GCS) score [19], MMSE score [20], pain on admission as measured subjectively, consumption of alcohol and tobacco prior to the MVA as determined in the structured interview, and other clinical information. These assessments were performed by a trained research nurse or psychiatrist.

To assess PTSD symptoms at follow-up, trained psychiatrists conducted the Clinician-Administered PTSD Scale (CAPS) at 1 and 6 months after the MVA injury. CAPS is the gold standard for assessing PTSD and involves a structured interview in which a Likert-type rating of frequency (0 to 4) and a separate rating of intensity (0 to 4) for each symptom is assigned [21]. To constitute a symptom, frequency must be ≥ 1 (once or twice a month) and intensity ≥ 2 (moderate). Full PTSD was diagnosed if patients fulfilled all of the symptom criteria (A-1, stressor; B, re-experiencing; C, avoidance; D, hyperarousal; E, duration; and F, impairment) according to the Diagnostic and Statistical Manual of Mental Disorders (DSM), Fourth Edition, Text Revision [22]. Partial PTSD was diagnosed if they fulfilled two of the three symptom criteria B, C, and D, and the criteria A-1, E, and F. A random sample of 30 patients assessed by two raters (YM and DN) was used to assess inter-rater reliability. The calculated intraclass correlation coefficient was 1.0.

#### Administration of propofol

The clinical practice guidelines of the American College of Critical Care Medicine recommend that propofol is administered for ongoing sedation within the first 3 days [13]; therefore, we retrospectively collected data from the medical records on the total dose of propofol over the initial 72-h period and the route of administration. We used propofol (2,6-disopropyl phenol) purchased from AstraZeneca Japan (Osaka, Japan).

#### Confounding factors

We investigated eight potential confounding variables, namely, female sex, age, ISS, GCS score, pain on admission, and administration of midazolam, morphine, or ketamine, for the following reasons. A meta-analysis revealed that civilian women were consistently at higher risk than men for PTSD [23]. In an epidemiological study, younger persons showed an increased risk of PTSD compared to elderly persons [24]. ISS was chosen as the objective accident-related variable, and GCS score was chosen as a surrogate marker of traumatic brain injury as more severe traumatic brain injury was found to be associated with a diminished risk of PTSD [25]. ISS and GCS score were determined by an emergency physician on admission. Some studies have indicated that the level of pain is significantly associated with subsequent risk of PTSD [26,27] and therefore we investigated whether or not patients complained of pain on admission.

The clinical practice guidelines of the American College of Critical Care Medicine recommend midazolam for short-term use (within 48 to 72 h) and lorazepam for sedation in most patients via intermittent intravenous (i.v.) administration or continuous infusion [13]. Benzodiazepines such as midazolam and lorazepam could affect PTSD symptoms by creating an amnesic effect [15,28,29]. Therefore, we checked administration of midazolam within 72 h of MVA, but not i.v. administration of lorazepam, which is not approved in Japan. Other studies have suggested that morphine administered in the acute stage of trauma may reduce the risk of subsequent development of PTSD [30,31]. Moreover, single-dose ketamine affected the severity and duration of posttraumatic stress symptoms in injured accident victims [32] and perioperative ketamine was associated with a lower prevalence of PTSD in burned soldiers than in those not receiving it [33]. Therefore, we also investigated the administration of morphine and ketamine within 72 h of MVA.

However, three potential covariates - GCS, pain on admission, and morphine administration - were excluded from the multivariate regression analysis because of zero, small, or sparse data for these covariates.

### Statistical analysis

We performed logistic regression analysis with diagnosis of full or partial PTSD at 6 months after the MVA as the primary outcome. Secondary outcomes were diagnosis of full or partial PTSD at 1 month and a CAPS score indicating PTSD at 1 month and 6 months after the MVA. We performed logistic regression analysis and calculated odds ratios (ORs) and 95% confidence intervals (95% CIs) for full and partial PTSD. Univariate regression analysis and multivariate regression analysis were used to examine the effect of using propofol on the CAPS score, accounting for the five potential covariates described above. Additionally, we calculated regression coefficients (β) and 95% CIs in the regression analysis as secondary outcomes. All analyses were performed using SPSS statistical software version 17.0J for Windows (SPSS, Tokyo, Japan).

As missing values were present at 1 and 6 months due to dropout and other reasons, we performed multiple imputations with SAS 9.1.3 via procedure MI (SAS System for Windows, version 9.1, SAS Institute, Cary, NC).

## Results

**Figure 1:**
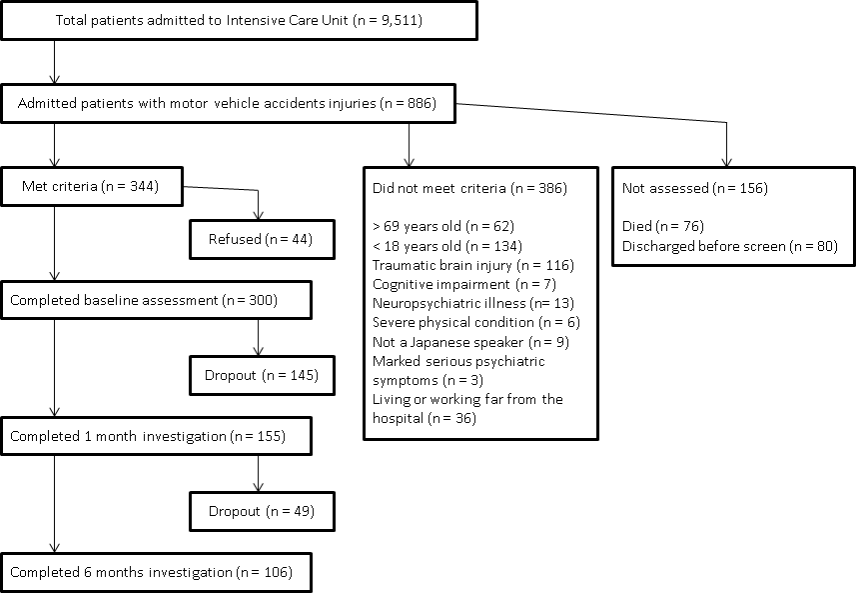
**Flow chart of enrollment and follow-up**.

The demographics and clinical characteristics of participants who were administered propofol within 72 h of an MVA are shown in Table 1. Sixty-seven (22.3%) were women and the mean age of all participants was 36.5 (SD 15.0) years. The median ISS was 8.0 (range 1 to 48) and the median GCS score was 15.0 (range 3 to 15). Twenty-six (9.7%) participants were administered propofol within 72 h of MVA, at an average dose of 942.2 mg (median 155 mg, 25th percentile 142.5 mg, 75th percentile 477.3 mg, range 60 to 6650 mg). Of the 300 participants, 155 (51.7%) completed the CAPS at the 1-month follow-up and 106 (35.3%) completed it at the 6-month follow-up (Figure 1). Reasons for dropout were as follows: refused to participate in follow-up (*n *= 24), no response to telephone and mail (*n *= 126), moved to an unknown address (*n *= 9), questionnaire data alone (*n *= 33), or excluded due to serious psychiatric symptoms (*n *= 2). None of the participants had died up to the 6-month follow-up. With the imputed data, the mean CAPS score was 18.2 points at 1 month and 14.0 points at 6 months. Ten participants (3.3%) were deemed to have full PTSD at 1 month and 8 (2.7%) were deemed to have full PTSD at 6 months [34]. Forty-two participants (14.0%) had full or partial PTSD at 1 month, with 21 participants (7%) having the same symptom level at 6 months.

**Table 1:** Demographics and clinical profile of participants critically injured in motor vehicle accidents

Clinical profile and administered drugs	Propofol used (*n *= 26)	Propofol not used (*n *= 274)	All participants (*n *= 300)
Age, years (range)	36.0 (18, 69)	36.6 (18, 65)	36.5 (18, 69)
Women, % (n)	23.1 (6)	22.3 (61)	22.3 (67)
Pain on admission, % (n)	88.5, 23	93.1, 255	92.7, 278
Glasgow Coma Scale score (range)	14.0 (3, 15)	14.6 (3, 15)	14.6 (3, 15)
Injury Severity Score (range)	18.1 (4, 41)	8.2 (1, 48)	9.1 (1, 48)
Heart rate on admission, bpm (range)	87.7 (52, 114)	84.7 (52, 140)	84.9 (52, 140)
History of self-reported psychiatric illness, % (n)	11.5 (3)	10.2 (28)	10.3 (31)
Smoker, % (n)	53.8 (14)	52.9 (145)	53.0 (159)
Alcohol consumption, % (n)			
Never drinker or past drinker	19.2 (5)	18.2 (50)	18.3 (55)
Occasional drinker/drinks 1 to 3 days/month	42.3 (11)	29.6 (81)	30.7 (92)
Drinks 1 to 2 days/week to almost every day	38.5 (10)	52.2 (143)	51.0 (153)
Midazolam used within 72 h, % (n)	50.0 (13)	8.0 (22)	11.7 (35)
Morphine used within 72 h, % (n)	11.5 (3)	1.0 (2)	1.7 (5)
Ketamine used within 72 h, % (n)	34.6 (9)	10.9 (30)	13 (39)

Table 2 shows that the participants who were administered propofol had a higher risk of meeting the criteria for full or partial PTSD at 1 month and 6 months after an MVA: on univariate logistic regression, the OR at 1 month was 2.52 (95% CI 0.99 to 6.42, *P *= 0.053) and at 6 months was 5.18 (95% CI 1.81 to 14.81, *P *= 0.002); on multivariate logistic regression, the OR at 1 month was 1.31 (95% CI 0.41 to 4.23, *P *= 0.647) and at 6 months was 6.13 (95% CI 1.57 to 23.85, *P *= 0.009).

**Table 2:** Multivariate analysis of propofol administration and diagnosis of full or partial posttraumatic stress disorder (PTSD) at 1 and 6 months after a motor vehicle accident

PTSD diagnosis		Logistic regression at 1 month			Logistic regression at 6 months		
		
	Covariates	OR	95% CI	*P*-value	OR	95% CI	*P*-value
**Full or partial**	Propofol	1.31	0.41, 4.23	0.647	6.13	1.57, 23.85	0.009
	Age	0.99	0.97, 1.01	0.397	1.04	1.00, 1.07	0.032
	Female	10.02	4.33, 23.17	< 0.001	5.76	1.97, 16.78	0.001
	ISS	1.10	1.05, 1.15	< 0.001	1.04	0.98, 1.11	0.217
	Midazolam	0.46	0.12, 1.69	0.240	0.40	0.06, 2.73	0.349
	Ketamin	1.91	0.62, 5.94	0.262	1.15	0.20, 6.51	0.878
**Full**	Propofol	0.90	0.09, 8.98	0.928	14.63	2.07, 103.29	0.007
	Age	1.04	0.99, 1.08	0.128	1.01	0.96, 1.06	0.819
	Female	7.53	1.65, 34.44	0.009	5.16	0.99, 27.04	0.052
	ISS	1.07	0.98, 1.17	0.112	0.94	0.82, 1.07	0.352
	Midazolam	0.42	0.02, 7.61	0.555	0.51	0.02, 12.08	0.673
	Ketamine	0.96	0.07, 14.00	0.974	1.19	0.06, 23.97	0.909

The independent variable was use of propofol within 72 h of a motor vehicle accident (MVA); *n *= 300 participants analyzed by multivariate logistic regression analysis of age, female sex, and administration of ketamine or midazolam within 72 h of an MVA. OR, odds ratio; ISS, injury severity score.

Table 3 shows the relationship between CAPS score at 1 month and 6 months after an MVA and administration of propofol. Univariate regression analysis revealed that participants who received propofol had more severe PTSD symptoms at 6 months (β = 4.84, *P *= 0.045, 95% CI 0.01 to 9.58), whereas multivariate regression analysis showed a non-significant trend toward adverse effects of propofol on PTSD symptom development (β = 4.08, *P *= 0.080, 95% CI -0.49 to 8.64).

**Table 3:** Propofol administration and Clinician-Administered posttraumatic stress disorder (PTSD) Scale score 1 and 6 months after a motor vehicle accident (MVA).

	Univariate regression			Multivariate regression		
		
	β	95% CI	*P-*value	β	95% CI	*P-*value
1 month	4.10	-1.67, 9.88	0.163	-0.42	-6.34, 5.51	0.890
6 months	4.84	0.10, 9.58	0.045	4.08	-0.49, 8.64	0.080

The independent variable was use of propofol within 72 h of an MVA; *n *= 300 participants analyzed by multivariate logistic regression analysis of age, female sex, and administration of ketamine or midazolam within 72 h of an MVA.

## Discussion

The findings of the present study suggest that the administration of propofol for MVA victims in the acute trauma stage could have adversely affected the later development of PTSD. In logistic regression analysis, use of propofol showed a higher risk for developing full or partial PTSD at 1 and 6 months after injury. However, the association seen on univariate logistic regression was not apparent on multivariate logistic regression at 1 month after MVA. The reasons for this lack of association on multivariate logistic regression might be explained by the confounding factor of physical condition (assessed by the ISS) and the clinical course. Some previous studies have suggested that injury-related PTSD symptoms tend to develop late [35,36]. For instance, 22% of MVA victims have been shown to meet DSM-III-R criteria for PTSD at 6 months after injury and 30% were diagnosed with PTSD at 12 months [35]. Further, 4.2% of US soldiers were found to have problematic PTSD at 1 month after injury compared with 12.0% at 7 months [36]. In the present study, in relation to secondary outcome, the β-value of regression analysis was over 4 points at 6 months after MVA. This value was comparable to having one further clinical PTSD symptom. This result suggests that propofol administration has adverse effects on not only categorical PTSD diagnosis but also continuous PTSD symptom level.

Several risk factors for PTSD have been reported in a meta-analysis [23]. However, few studies have examined whether propofol is one of these risk factors, and in this respect our findings offer new insight when considering the risk factors for PTSD. Our results are similar to those of Hauer *et al. *in that propofol enhanced consolidation of retrograde fear memory in rats [16]. They suggested that enhancement of retrograde memory by propofol depends on an indirect activation of CB1 cannabinoid receptors [16]. Their hypothesis was based on the fact that administration of propofol indirectly increases anandamide (one of the main endocannabinoids) within the mouse brain by inhibiting fatty acid amide hydrolase, which degrades endocannabinoids [37]. It is well known that endocannabinoids adversely affect a hippocampal-dependent memory task [38]. However, recent studies have demonstrated that endocannabinoids could facilitate emotionally charged memories [38,39]. Accordingly, the formation of emotional memory is similar to the development of PTSD. Patel *et al. *showed that other intravenous general anesthetics, including midazolam, ketamine, etomidate, and thiopental, did not affect fatty acid amide hydrolase activity at sedative-relevant concentrations [37]. This also raises the possibility that propofol specifically facilitated formation of retrograde fear memory in our study by excluding the effect of other sedative drugs. In addition, our findings might support the previous observation that propofol use in the ICU was associated with delusional memory and PTSD symptoms after discharge [40].

The present study has several limitations. First, the observational design of this study meant we could not divide patients into two groups in regard to the use of propofol and the sample size of patients who were administered propofol was modest. Second, previous studies have pointed out that the length of ICU stay is a predictor of PTSD, a factor we did not investigate due to insufficient data. Third, we wanted to adjust for three potential covariates, namely GCS, pain on admission, and morphine administration, in the multivariate analysis, but could not do so due to data problems. Further studies of these potential confounders are warranted. Fourth, the response rate was low, especially at 6 months. Weisaeth had pointed out that the high potential loss to follow-up would reduce the predictor value, thus the response rates need to be high in a longitudinal trauma study [41]. In addition, the risk of either type I or II errors increase as time passes. We should evaluate the present results with these points in mind.

## Conclusions

The findings of this study indicate that administration of propofol during the acute trauma stage after an MVA may adversely affect the development of later PTSD. However, a retrospective design tends to overestimate the treatment effects, and therefore the findings should be interpreted with caution. Clearly these results are not definitive, but they do provide supplementary evidence to animal studies and could be helpful for emergency physicians to consider. Studies on PTSD risk factors as a result of drug administration for trauma are still relatively few; in particular, the effects of anesthetic drugs on fear memory remained to be investigated. To clarify the correlation between the administration of sedative drugs and development of PTSD, further collaborative research from bench to bedside, and between critical care medicine and psychiatry are needed.

## Key messages

• Modulating the formation of fear memory after trauma is an important strategy to prevent the subsequent development of PTSD symptoms.

• Administration of propofol within 72 h of trauma could adversely affect the development of PTSD symptoms at 6-month follow-up.

• Propofol administration was shown to have adverse effects not only on categorical PTSD diagnosis, but also on continuous PTSD symptom levels.

• The present results support the findings that propofol enhances consolidation of retrograde fear memory via the endocannabinoid system.

## Abbreviations

CAPS: Clinician-Administered PTSD Scale; DSM: Diagnostic and Statistical Manual of Mental Disorders; GCS: Glasgow Coma Scale; ICU: intensive care unit; ISS: injury severity score; i.v.: intravenous; MMSE: Mini-Mental State Examination; MVA: motor vehicle accident; NDMC: National Disaster Medical Center; PTSD: posttraumatic stress disorder.

## Competing interests

There are no competing interests to disclose related to this manuscript.

Other financial disclosures are as follows: MU has no financial competing interests; YM has received research support from the Japan Science and Technology Agency, CREST and the Ministry of Health, Labor, and Welfare of Japan, Intramural Research Grant for Neurological and Psychiatric Disorders of NCNP and lecture fees from Suntory Wellness Ltd., Eli Liliy Japan KK, and Otsuka Pharmaceutical Co., Ltd. DN has received research support from Toray Industries, Inc. and the Foundation for Total Health Promotion, and lecture fees from Qol Co., Ltd, DHA & EPA Association and NTT DoCoMo, Inc. NY has received research support from the Japan Society for the Promotion of Science and the Japanese Ministry of Health, Labor and Welfare. KM has received research support from the Japan Society for the Promotion of Science, the 26th Research Grant in Medical and Health Science of Meiji Yasuda Life Foundation of Health and Welfare. YO has research support from the Japanese Ministry of Health, Labor, and Welfare. YK has received research support from the Japanese Ministry of Health, Labor, and Welfare, Japan Science and Technology Agency, CREST, and received a grant from GlaxoSmithKline, Japan; he has been a speaker for GlaxoSmithKline, Pfizer, Meiji Seika Pharma, Yoshitomi Pharmaceutical and Meiji Yasuda Insurance Co. SK has received a grant from GlaxoSmithKline, Pfizer and Shionogi Pharmaceutical Co. and received research support from the Ono Pharmaceutical Co.; he has been a speaker for Mochida Pharmaceutical, Astellas, GlaxoSmithKline, Asahi Kasei Pharma and Mitsubishi Tanabe Pharma Co.

## Authors' contributions

MU designed this secondary analysis, participated in data collection, performed statistical analysis and wrote the manuscript. YM conceived the original cohort study, wrote the protocol, recruited and interviewed the participants and revised the manuscript critically for important intellectual content. DN joined the preparation of the original protocol, recruited and interviewed the participants and revised the manuscript critically for important intellectual content. NY performed statistical analysis for multiple imputations and reviewed the study design and statistical analysis. KM performed interpretation of data and revised the manuscript critically for important intellectual content. YO and YK joined the preparation of the original protocol and revised the manuscript critically for important intellectual content. SK has made contributions to analysis and interpretation of data and revised the manuscript critically for important intellectual content. All authors read and approved the final manuscript.
